# Associations of *miR-181a* with Health-Related Quality of Life, Cognitive Functioning, and Clinical Data of Patients with Different Grade Glioma Tumors

**DOI:** 10.3390/ijms231911149

**Published:** 2022-09-22

**Authors:** Indre Valiulyte, Aiste Pranckeviciene, Adomas Bunevicius, Arimantas Tamasauskas, Hanna Svitina, Inessa Skrypkina, Paulina Vaitkiene

**Affiliations:** 1 Laboratory of Molecular Neurooncology, Neuroscience Institute, Lithuanian University of Health Sciences, Eiveniu Str. 4, LT-50161 Kaunas, Lithuania; 2 Laboratory of Behavioral Medicine, Neuroscience Institute, Lithuanian University of Health Sciences, Eiveniu Str. 4, LT-50161 Kaunas, Lithuania; 3 Laboratory of Biosynthesis of Nucleic Acids, Department of Functional Genomics, Institute of Molecular Biology and Genetics of NASU, Zabolotnogo Str. 150, 03143 Kyiv, Ukraine; 4 Laboratory of Molecular Biology, Neuroscience Institute, Lithuanian University of Health Sciences, Eiveniu Str. 4, LT-50161 Kaunas, Lithuania

**Keywords:** miR-181a, glioma, GBM, IDH1, survival, health-related quality of life

## Abstract

Gliomas are central nervous system tumors with a lethal prognosis. Small micro-RNA molecules participate in various biological processes, are tissue-specific, and, therefore, could be promising targets for cancer treatment. Thus, this study aims to examine *miR-181a* as a potent biomarker for the diagnosis and prognosis of glioma patients and, for the first time, to find associations between the expression level of *miR-181a* and patient quality of life (QoL) and cognitive functioning. The expression level of *miR-181a* was analyzed in 78 post-operative II-IV grade gliomas by quantitative real-time polymerase chain reaction. The expression profile was compared with patient clinical data (age, survival time after the operation, tumor grade and location, mutation status of isocitrate dehydrogenase 1 (*IDH1*), and promoter methylation of O-6-methylguanine methyltransferase). Furthermore, the health-related QoL was assessed using the Karnofsky performance scale and the quality of life questionnaires; while cognitive assessment was assessed by the Hopkins verbal learning test-revised, trail-making test, and phonemic fluency tasks. The expression of *miR-181a* was significantly lower in tumors of grade III and IV and was associated with *IDH1* wild-type gliomas and a worse prognosis of patient overall survival. Additionally, a positive correlation was observed between *miR-181a* levels and functional status and QoL of glioma patients. Therefore, *miR-181a* is a unique molecule that plays an important role in gliomagenesis, and is also associated with changes in patients’ quality of life.

## 1. Introduction

Astrocytic origin gliomas (astrocytomas) are central nervous system (CNS) tumors. According to the World Health Organization’s (WHO) 2016 classification of CNS tumors, these tumors are graded as grade II—diffuse astrocytoma, grade III—anaplastic astrocytoma, and grade IV—glioblastoma (GBM), of which GBM is the most common and malignant primary brain tumor [1]. The treatment of GBM remains a challenge. Despite available treatment options (surgical resection, adjuvant radiotherapy, and chemotherapy), the average life expectancy of patients diagnosed with GBM is only slightly above one year [2,3]. The dismal patient outcome is due to a tumor’s ability to regrow (~90%) and molecular heterogeneity [4]. Genomic and transcriptome studies revealed that histologically identical GBM forms may belong to different molecular subtypes, leading to different responses to treatment and patient life expectancy [5,6,7,8]. Therefore, more precise molecular identification of gliomas is necessary to prescribe more effective individualized therapies that would prolong patients’ survival times. This strategy is already widely used in the diagnosis of various other oncological diseases [9,10]. Nevertheless, the increased life expectancy of patients should not be the only aim of improved GBM therapies; quality of life with the oncological disease is of equal importance. Research indicates that the quality of life of GBM patients remains extremely poor [11]. Therefore, it is important to discover novel molecules that could more accurately predict the behavior of the tumor and the patient’s quality of functioning after the surgery.

In recent years, it was demonstrated that micro RNAs (miRNAs) are associated with tumor progression and drug resistance by targeting genes associated with drug resistance or by affecting genes involved in cancer cell proliferation, cell cycle, and apoptosis [12,13,14]. Mature miRNAs are short, non-coding, regulatory RNAs of 21–25 nucleotides involved in the post-transcriptional regulation of gene expression by binding to the 3′ UTR of an mRNA. According to numerous studies, miRNAs regulate about a third of human genes and are involved in many biological processes, such as nervous system regulation, angiogenesis, cell cycle control, cell differentiation, proliferation, apoptosis, and even the immune response [15,16]. Importantly, a single miRNA targets many genes, has a high specificity for tissue, and is sensitive to tumor progression. As a result, depending on the organ or tissue, miRNA molecules may act as inhibitors and/or oncogenes and could be used as a non-invasive way to diagnose and predict disease [17].

One of the most studied and promising biomarkers with predictive value for the prognosis of cancer progression is *miRNA-181a*, which belongs to the miR-181 family. The family of miR-181 is composed of four different mature forms, namely *miR-181a*, *miR-181b*, *miR-181c*, and *miR-181d*, localized to three separate chromosomes (1, 9, and 19) [18]. The research studies have reported the involvement of *miR-181a* in diverse cellular functions such as cell growth, proliferation, death, survival, and maintenance, as well as gliomagenesis [18,19,20]. Therefore, the idea of this study is to examine *miR-181a* as a potent biomarker for the diagnosis and prognosis of glioma patients and to find associations between the expression level of *miR-181a* and patients’ health-related symptoms.

## 2. Results

### 2.1. Association of miR-181a Expression with Patient Clinicopathological Data

**Figure 1:**
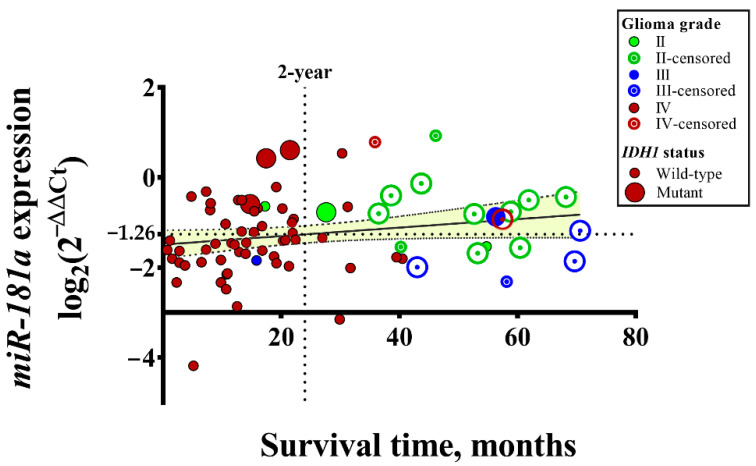
Distribution of patients with the diagnosis of different malignancy grade astrocytoma tumors, according to patient survival time and expression of *miR-181a*. The average of *miR-181a* mRNA expression is −1.26 (dotted line in the *y*-axis). Pearson correlation r = 0.206, *p* = 0.071, yellow area represents 95% confidence interval.

To reveal the importance of *miR-181a* in the pathogenesis of astrocytomas, the mRNA expression of *miR-181a* was analyzed in 78 different malignancy grade tumors. In Figure 1, it was demonstrated that the gene expression was diversified. The majority of GBM patients with lower than average (<−1.26) mRNA expression died within 2 years, while patients with lower-grade tumors had a higher expression of *miR-181a* and survived 2 to 6 years.

**Figure 2:**
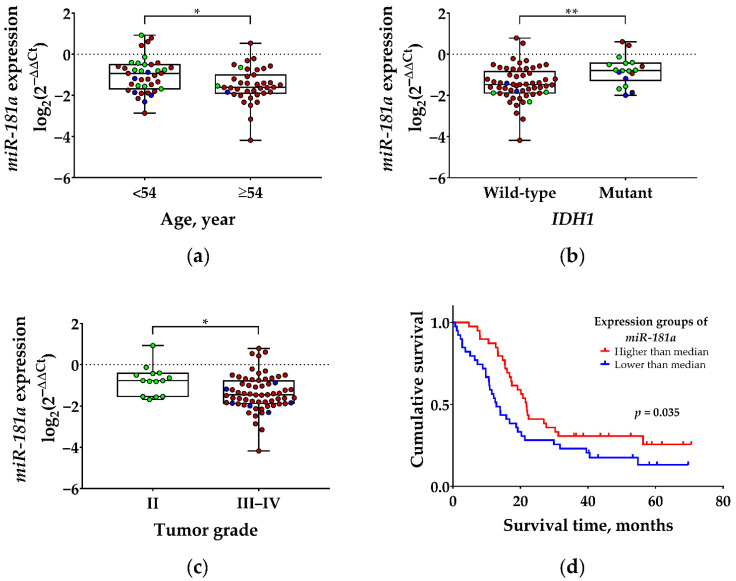
Statistically significant associations of *miR-181a* expression with patient (**a**) age; (**b**) *IDH1* status; (**c**) tumor grade; (**d**) patient survival time. The boxplots indicate mean, within 25 and 75 percentiles, min, and max values. Green dots—grade II, blue—III, and red—IV (GBM). Student’s *t*-test, * *p* < 0.05, ** *p* < 0.01.

As follows, to determine whether expression changes of *miR-181a* were significantly associated with patient clinicopathological characteristics, *miR-181a* expression was divided into “low” (<mean of *miR-181a* mRNA expression) and “high” (≥mean) gene expression groups. The analysis reveals that a higher expression of *miR-181a* is significantly associated with younger age of patients (<54-year, *p* = 0.006), lower tumor malignancy grade (*p* = 0.036), and gliomas with a mutant-type of *IDH1* (*p* = 0.002) (see Table 1; Figure 2a–c). According to the Kaplan–Meier analysis, patients with a higher expression of *miR-181a* have a significantly higher chance of longer survival, compared with patients with low gene expression values (Log-rank test, χ^2^ = 4.465, df = 1, *p* = 0.035) (see Figure 2d). The median survival time was 8.9 months longer in the patient group with higher expression of *miR-181a*.

The univariate cox regression analysis reveals that patients’ clinical characteristics such as age, tumor malignancy grade, and *IDH1* status, as well as the expression of *miR-181a*, are significantly associated with patient overall survival (OS). However, according to the multivariate cox regression analysis, the tumor stage and *IDH1* status are the only covariates significantly associated with the OS of glioma patients (see Table 2).

### 2.2. Associations of miR-181a Expression with IDH1 Status of GBM Tumors

**Figure 3:**
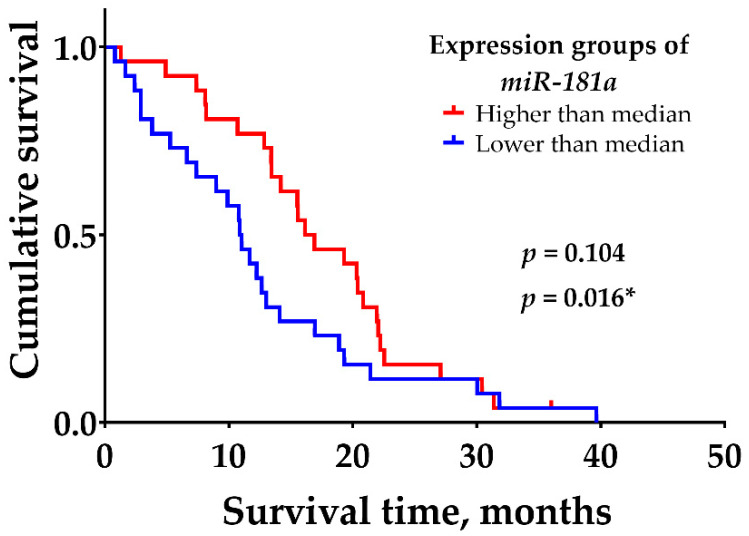
Survival analysis of patients with the diagnosis of *IDH1* wild-type GBM in higher and lower *miR-81a* expression groups. *p*-values of log-rank test and * Gehan–Breslow–Wilcoxon test.

In addition, it was noted that all *IDH1* mutant GBM tumors were detected in the higher *miR-181a* mRNA expression group, and the majority of *IDH1* wild-type GBM tumors (62%, 32/52) were detected in the lower gene expression group (see Figure 1 and Figure 2b). According to Student’s *t*-test, the noted difference was statistically significant (*p* = 0.005). To find out whether *miR-181a* expression was associated with patient survival, only *IDH1* wild-type GBM tumors were selected and divided into two groups, according to the median of *miR-181a* expression. Kaplan–Meier analysis shows the tendency that patients with a higher expression of *miR-181a* have a significantly higher chance of longer survival, compared with patients with low gene expression values (see Figure 3). According to the log-rank test, the difference was not statistically significant (χ^2^ = 2.64, df = 1, *p* = 0.104); however, the Gehan–Breslow–Wilcoxon method, which gives more weight to deaths at early time points, shows a statistically significant difference (χ^2^ = 5.83, df = 1, *p* = 0.016).

### 2.3. Associations of mir181a Expression with Functional Status, QoL, and Cognitive Functioning of Patients

The correlation analysis was performed to reveal *miR-181a* expression associations with variables related to the quality of functioning (see Table 3). The results reveal that the expression of *miR-181a* positively correlates with general quality of life (EORTC QLQ-C30; r = 0.310, *p* = 0.010) and functional status, evaluated by a clinician (KPS; r = 0.237, *p* = 0.049) in all glioma patients. Interestingly, a statistically significant positive correlation between *miR-181a* and a better quality of life was reflected in the group of men (*p* = 0.009) rather than women (*p* = 0.469). In addition, as patients with GBM experience more severe symptoms, it was decided to analyze this subgroup separately. The expression levels of *miR-181a* show a significantly positive correlation with patient quality of life (EORTC QLQ-C30; r = 0.290, *p* = 0.041), but an inverse correlation with patient memory (HVLT-R; r = −0.291, *p* = 0.040). However, no statistically significant correlations of *miR-181a* with tumor-related symptoms (EORTC QLQ-BN20), depression (PHQ-9), cumulative learning (HVLT-R), psychomotor speed (TMT-A), executive functioning (TMT-B), and/or verbal fluency were determined.

## 3. Discussion

Numerous studies have shown that miRNAs, which regulate biological processes such as cell proliferation, apoptosis, metabolism, and/or differentiation, are thought to have clinical potential in cancer prognosis and treatment [21]. Among the so-far-characterized miRNAs, *miR-181a* is involved in several types of cancer [18]. A significant upregulation of *miR-181a* level has been found in breast cancer [22], ovarian cancer [23], pancreatic cancer [24], hepatocellular carcinoma [25], and oral squamous cell carcinoma [26], whereas evident downregulation of *miR-181a* has been detected in non-small cell lung cancer [27] and prostate cancer [28], as compared with healthy controls. Our study also reveals that the expression levels of *miR-181a* decrease during astrocytoma progression. Comparable results were observed by other researcher groups, which demonstrates the downregulation of *miR-181a* in all grade glioma tumors (WHO II–IV), GBM cell lines, and glioma stem cells (GSCs), as compared with normal brain tissue, astrocytes, and differentiated GBM cells, respectively [29,30,31,32]. Importantly, the overexpression of *miR-181a* inhibited proliferation, migration, invasion, epithelial-mesenchymal transition (EMT), and induced apoptosis of GBM cells [30,33,34]. The process of apoptosis was modulated by targeting the apoptosis-related genes (p53, Bax, Bcl-2, Bim, etc.) [19], while proliferation was modulated by the downregulation of the MAPK pathway [35]. In addition, the upregulation of *miR-181a* sensitized the GBM cells to temozolomide (TMZ) and radiation treatment [34,36] also suppressed the formation of GSCs and inhibited GBM tumorigenesis [31]. More importantly, the study by Wu et al. showed the clinical significance of circulating *miR-181a* in patients with glioma tumors. Before the operation, circulating *miR-181a* was found to be downregulated in the plasma of GBM patients as compared with lower-grade tumors [37]. After 10 days, the levels of *miR-181a* increased more than 10-fold. In addition, a lower expression of circulating *miR-181a* was significantly associated with poor OS [37], as was demonstrated in our study with astrocytoma tumors. Therefore, the aberrantly downregulated *miR-181a* could be a critical factor that contributes to the malignant appearance of astrocytoma.

Our study also demonstrates a significant association between the expression levels of *miR-181a* and patient age, as well as *IDH1* status. However, according to the multivariate Cox regression analysis, only the tumor grade and *IDH1* mutation were the best predictors of patient OS in our study cohort. Importantly, we noted that the mutant form of *IDH1*, which is the factor of a good astrocytoma patient survival prognosis, was found in GBM tumors with an increased expression of *miR-181a*. All these GBM patients with *IDH1* mutation survived more than 14 months, and one of them survived even more than 57 months. We hypothesize that the expression of *mirR-181a* may influence the activity of the IDH1 protein which affects several major metabolic processes of the cells [38] and, therefore, has an impact on the OS of GBM patients. This relation between *IDH1* and *miR-181a* in GBM patients was also observed by Sippl et al. [39]. They determined the inverse correlation between the expression of *miRNA-181a2* and mRNA expression of *IDH1* (*p* = 0.06, r = −0.55). Nevertheless, studies on a larger sample would be needed to confirm our observations.

Next, we wanted to find out whether the survival of patients with *IDH1* wild-type GBM tumors depends on *miR-181a* expression level. Since the survival of GBM patients after surgery is generally short, we additionally used the Gehan–Breslow–Wilcoxon method, which gives more weight to deaths at early time points. The analysis demonstrates that patients with a lower expression of *miR-181a* have significantly worse OS as compared with those with a higher gene expression level. However, the opposite effect was noted by another research group. They demonstrated that in patients with *IDH1* wild-type GBMs, low *miR-181a2* expression correlated with a prolonged OS (*p* = 0.019), [39]. The discrepancy may have occurred because different *miR-181a* isoforms were used in the analyses, which might have distinct biological functions. In our study, we analyzed the *miR-181a1* isoform, which is located on chromosome 1 (37.p5), while *miR-181a2* is situated on chromosome 9 (37.p5) [18]. Both isoforms produce almost identical mature *miR-181a*, but could be regulated by distinct molecules and, therefore, be differently expressed. For example, in human blood natural-killer cells, *pri-miR-181ab-2* levels were higher than *pri-miR-181ab-1*. The immunosuppressive cytokine, TGF-β, suppressed *pri-miR-181ab-1* expression while elevating *pri-miR-181ab-2* expression. On the contrary, interleukins −2, −15, and −12/−18 increased the expression of *pri-miR-181ab-1*, but inhibited *pri-miR-181ab-2* [40].

To the best of our knowledge, this was the first study comparing expression levels of *miR-181a* with patient quality of life. The analysis reveals that *miR-181a* positively correlates with the general quality of life subjective reported by patients themselves, as well as functioning status, evaluated by the treating clinician. However, no statistically significant correlations were found between *miR-181a* expression and more specific tumor-related symptoms, levels of depression, or cognitive functioning. A relatively small sample size, especially of tumor grades II and III, could affect the statistical power of our analysis. Furthermore, cognitive impairment and the profile of tumor-related symptoms are highly dependent on the localization of the tumor and other clinical factors, such as edema, tumor-induced compression to nearby tissues, or the frequency of seizures. Thus, it might be that relationships were lost due to the heterogeneity of the sample regarding tumor locations and other clinical characteristics. However, previous findings by our team group showed that miR-34a or *miR-181b*/*d* expression levels were related to patients’ functioning and tumor-related symptoms [41,42]. Therefore, we believe that current findings support the idea that levels of *miR-181a* expression might be valuable not only in predicting longer survival but also in better general functioning. Still, further studies are needed to clarify the mixed findings in male and female subgroups.

## 4. Materials and Methods

### 4.1. Samples and Patient Clinicopathological Data

Seventy-eight samples from patients with the diagnosis of II-IV grade glioma tumors were analyzed in the study. All patients underwent neurosurgery at the Department of Neurosurgery, Hospital of Lithuanian University of Health Sciences, from 2015 to 2018. After surgical resection, tumor samples were frozen in liquid nitrogen. The diagnosis was confirmed by the pathologists. The study was approved by the Kaunas Regional Biomedical Research Ethics Committee, and written patient consent was taken from each patient before inclusion in the study.

The clinical data, such as gender, age at the time of surgery, tumor grade, isocitrate dehydrogenase 1 (*IDH1*) status (the R132H mutation in the *IDH1* gene), and methylation of O-6-methylguanine methyltransferase (*MGMT*) were collected from medical records. According to the WHO classification 2016 [1], there were 14 tumor samples of grade II, 6 of grade III, and 58 of grade IV (GBM). There were 35 women and 43 men, with a mean age of 54 years (range: 24–80 years). The overall survival of the patient was calculated from the date of tumor resection to the date of patient death or database closure (5 October 2021).

### 4.2. Functional Status

Patient functional status was assessed by a treating neurosurgeon during the hospital stay using the Karnofsky performance scale (KPS) [43]. The KPS measures a patient’s ability to carry on his/her normal daily activities and dependence on help and nursing care using an 11-point rating scale. The total KPS score ranges from 100 (normal functioning) to 0 (death), with higher scores indicating better daily functioning and higher functional independence. Data on functional status was available for 70 (88.6%) of patients.

### 4.3. Quality of Life Assessment

Quality of life (QoL) assessment was performed 2–3 days before the neurosurgery. Patients were asked to fill out self-report questionnaires addressing their symptoms and quality of life. If assistance was needed due to reading, motor, or visual problems, questionnaires were filed with the help of a medical psychologist. Data on QoL was available for 68 (87.2%) of the patients.

The European Organization for Research and Treatment of Cancer quality of life questionnaires QLQ-30 [44] and QLQ-BN20 [45] for brain tumor-related symptoms were used in this study. The QLQ-C30 contains 30 items that were designed to assess global health status, subjectively reported functional status, role functioning, emotional functioning, cognitive functioning, social functioning, and various cancer-related symptoms. Raw scores were linearly transformed to 0–100 scales, with higher scores indicating better quality of life.

The QLQ-BN20 is a 20-item self-rating scale specifically developed for the assessment of health complaints in brain tumor patients. The questionnaire contains many common BT-related symptoms, including future uncertainty, visual disorder, cognitive impairment, etc. The QLQ-BN20 scores were linearly transformed to a 0–100 scale, with a higher score indicating greater BT-related symptom severity.

In addition to QoL instruments, we also included a measure of depression, as depression is significantly related to decreased QoL in many patient populations. The patient health questionnaire-9 (PHQ-9) [46] was chosen for the assessment of current depressive symptoms. The PHQ-9 is based on the diagnostic statistical manual-IV depression diagnostic criteria, and it is recognized as a valid and reliable tool for depression screening in glioma patients [47,48].

### 4.4. Assessment of Cognitive Functioning

A cognitive assessment was performed by a medical psychologist 2–3 days before the neurosurgery at the Department of Neurosurgery of the Hospital of LUHS. A set of neuropsychological tests, recommended for the assessment of treatment outcomes in glioma studies, was used [49].

The Hopkins verbal learning test-revised (HVLT-R) was used for verbal memory assessment [50]. The test consists of 12 words that are read aloud for three trials, each trial followed by a patient’s free recall. After an approximately 20 min. delay, during which other tests are administered, the patient is asked to recall the list of words. Two scores: cumulative learning (total number of words recalled in trials 1, 2, and 3) and delayed recall (number of words recalled after a delay) were analyzed in this study.

The Trail-making test (TMT, parts A and B) was used for the assessment of psychomotor speed and executive functioning [51]. During the task, a patient is asked to connect a sequence of 25 targets (numbers 1, 2, 3, etc. in Part A, and numbers and letters in Part B) on a sheet of paper. The time of completion (in seconds) is considered as an indicator of psychomotor speed and executive functions.

Verbal fluency was measured using phonemic fluency tasks [52]. Patients were asked to produce as many words as possible beginning with a specific letter within the one-minute interval. Three trials using the letters K, A, and S were performed. The total number of words produced during three trials is used as a verbal fluency indicator.

As cognitive performance is sensitive to aging processes, all primary scores of neuropsychological tests were transformed to age-adjusted T scores (Mean 50, SD 10) using available norms for the Lithuanian population, with higher scores indicating better cognitive function. Memory and verbal fluency data were available for 68 (87%) of the patients, and data on psychomotor speed and executive function was available for 62 (79.5%) of the patients, as some patients were not able to perform these tasks due to visual or motor impairment.

### 4.5. Gene Expression Analysis

Small RNAs were extracted from frozen tumor tissue using a mirVana™ miRNA Isolation Kit (Life Technologies, Carlsbad, CA, USA, cat. no. AM1560), according to the manufacturer’s instructions. The quality and concentration were determined by NanoDrop 2000 (Thermo Fisher Scientific, Wilmington, DE, USA). Following this, 10 ng of purified micro RNAs were synthesized to cDNA using the “TaqMan Advanced miRNA cDNA Synthesis Kit” (Thermo Fisher Scientific, Pleasanton, CA, USA, cat. no. A25576).

The RT-PCR with TaqMan™ Fast Advanced Master Mix (Life Technologies, Carlsbad, CA, USA, cat. no. 4444965) was performed to analyze *miR-181a* expression changes in II-IV grade glioma tumors and healthy human brain RNA sample “FirstChoice Human Brain Reference RNA” (RHB; Ambion, Austin, TX, USA, cat. no. AM7962). The reaction consisted of 6 µL of TaqMan^®^ Fast Advanced Master Mix, 0.6 µL of hsa-miR-181a-3p probe (Applied Biosystems, Foster City, CA, USA, Assay ID: 479405_mir), 3 µL of cDNA sample, and nuclease-free water to a total volume of 12 µL. The reactions were performed in the RT-PCR System “Applied Biosystems 7500 Fast” (Applied Biosystems, Foster City, CA, USA) using a fast-cycling program. In addition, the housekeeping genes has-miR191-5p (Assay ID: 477952_mir), has-miR361-5p (Assay ID: 478056_mir), has-miR345-5p (Assay ID: 478366_mir), and has-miR103a-3p (Assay ID: 478253_mir) were measured to normalize the data. The average of the housekeeping genes was used for the comparative 2^−∆∆Ct^ method, in which gene expression in tumor samples was compared to healthy brain tissue.

### 4.6. Statistical Analysis

Statistical programs SPSS (version 25.0, IBM SPSS, Armonk, NY, USA) and GraphPad Prism (version 7.0, Graph-Pad Software, San Diego, CA, USA) were used for data analysis. The normality was confirmed by the Kolmogorov–Smirnov test. The Student’s independent *t*-test was applied to evaluate the difference in *miR-181a* expression between the two groups. Meanwhile, one-way ANOVA with Tukey’s correction was used for the three groups. The Chi-square test was used for the comparison of categorical variables and the Pearson correlation was used for the quantitative variables. Patient survival was evaluated according to the Kaplan–Meier curves with log-rank or Breslow tests. Univariate and multivariate (with the backward conditional method) Cox regression analyses were used to evaluate the relationships between clinical, psychological, and molecular variables and patient overall survival. The significance level was defined as *p* < 0.05.

## 5. Conclusions

**Figure 4:**
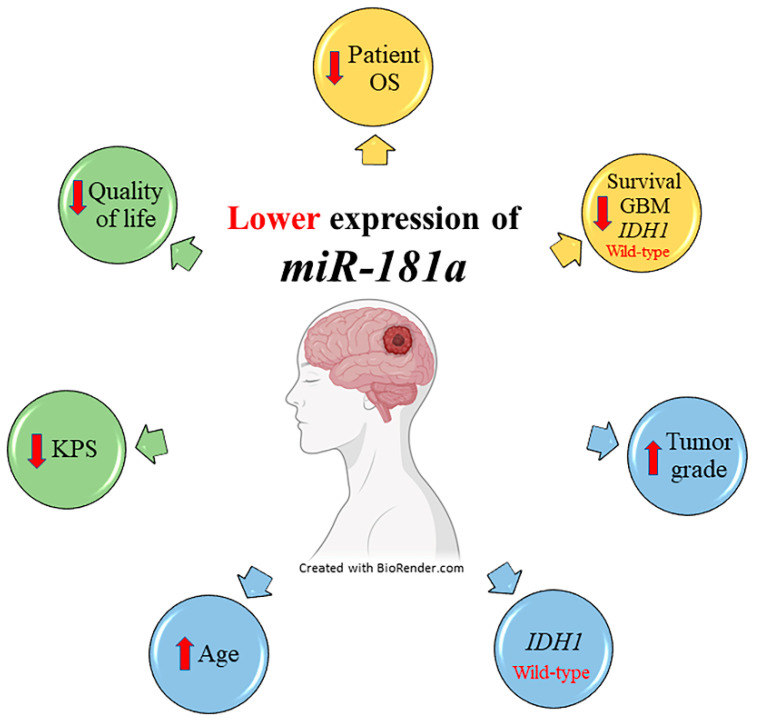
Statistically significant associations between expression of *miR-181a* and patient clinical data and psychology. KPS—Karnowski performance scale. The up arrow indicates higher tumor grade and older patient age, while down arrow—shorter patient survival time, lower KPS index, and worse patient quality of life.

The study suggests that *miR-181a* could be a promising biomarker for glioma patients, since the expression of *miR-181a* decreases during tumor progression and the downregulation of the gene is significantly associated with the worst patient survival prognosis. More importantly, lower expression levels of *miR-181a* are also related to wild-type *IDH1*, older age, as well as worse patient quality of life and functioning (see Figure 4).

However, the findings of this study should be seen in light of some limitations. One of them is a small number of tumor samples and incomplete sample information. Unfortunately, not all of the patients had information about *IDH1*, *MGMT* status, and functional data. Furthermore, patients’ genetic and medical histories were not considered during this study, which might affect the evaluation of patients’ health-related quality of life. This may have affected the reliability of the results. Therefore, follow-up studies are needed to confirm the prognostic value of *miR-181a* in glioma patients.

## Figures and Tables

**Table 1:** The relationships between gene expression of *miR-181a* and patient clinical characteristics.

Variables	Total No.	Expression of *miR-181a*		
		Low (%)	High (%)	*p*-Value
**Gender**				
Male	43	20 (46.5)	23 (53.5)	0.15
Female	35	22 (62.9)	13 (37.1)	
**Age, year**				
<54	39	15 (38.5)	24 (61.5)	**0.006**
≥54	39	27 (69.2)	12 (30.8)	
**Grade**				
II	14	4 (28.6)	10 (71.4)	**0.036**
III–IV	64	38 (59.4)	26 (40.6)	
***MGMT***				
Unmet	34	19 (55.9)	15 (44.1)	0.978
Met	36	20 (55.6)	16 (44.4)	
***IDH1***				
Wt	57	36 (63.2)	21 (36.8)	**0.002**
Mut	18	4 (22.2)	14 (77.8)	
**Tumor location**				
Right hemisphere	37	23 (62.2)	14 (37.8)	0.25
Left hemisphere	37	18 (48.6)	19 (54.4)	
Bilateral	4	1 (25.0)	3 (75.0)	

Unmet—unmethylated, Met—methylated, Wt—wild-type, Mut—mutant. Significant associations *p* < 0.05 indicated in bold numbers.

**Table 2:** Univariate and multivariate Cox regression analysis of clinicopathological variables and expression of *mir-181a*.

Variables	Univariate		Multivariate	
	HR (95% CI)	*p*-Value	HR (95% CI)	*p*-Value
**Gender**	0.732 (0.441–1.214)	0.226	N/A	
Female vs. Male				
**Age, year**	3.856 (2.205–6.741)	**<0.001**	1.417 (0.778–2.581)	0.254
<54 vs. ≥54				
**Grade**	9.748 (3.029–31.373)	**<0.001**	2.921 (1.459–5.846)	**0.002**
II vs. III–IV				
***MGMT***	0.712 (0.417–1.213)	0.211	N/A	
Unmeth vs. Meth				
***IDH1***	0.105 (0.041–0.272)	**<0.001**	0.314 (0.100–0.989)	**0.048**
Wt vs. Mut				
**Expression of *miR-181a***	0.584 (0.350–0.974)	**0.039**	0.909 (0.521–1.585)	0.737
Low vs. High				

Unmeth—unmethylated, Meth—methylated, Wt—wild-type, Mut—mutant, N/A—not applicable, HR—hazard ratio. Significant associations *p* < 0.05 indicated in bold numbers.

**Table 3:** Relationship between health-related quality of life indicators, clinical evaluation of patient’s functioning, and expression of *miR-181a* in different malignancy grade glioma patients.

Variables	Total			Female			Male			GBM		
	N	r	*p*	N	r	*p*	N	r	*p*	N	r	*p*
**EORTC QLQ-C30** Quality of Life	68	0.310	**0.010**	31	0.135	0.469	37	0.423	**0.009**	50	0.290	**0.041**
**EORTC QLQ-BN20** Tumor Related Symptoms	68	−0.209	0.088	31	−0.005	0.978	37	−0.311	0.061	50	−0.223	0.120
**PHQ-9** Depression	68	−0.128	0.299	31	0.060	0.750	37	−0.211	0.210	50	−0.126	0.385
**KPS** Functional status evaluated by a clinician	70	0.237	**0.049**	33	0.041	0.822	37	0.291	0.080	54	0.260	0.057
**HVLT-R** Cumulative learning	68	−0.175	0.155	32	−0.102	0.580	36	−0.194	0.256	50	−0.245	0.087
**HVLT-R** Delayed recall	68	−0.226	0.064	32	−0.094	0.607	36	−0.282	0.096	50	−0.291	**0.040**
**TMT-A** Psychomotor speed	63	0.010	0.941	28	−0.218	0.265	35	0.112	0.523	45	0.026	0.866
**TMT-B** Executive functions	63	0.079	0.538	28	−0.240	0.218	35	0.224	0.197	45	0.031	0.838
**Verbal fluency**	68	−0.134	0.276	32	−0.213	0.241	36	−0.078	0.652	50	−0.167	0.245

r—Pearson correlation coefficient. EORTC QLQ-30—The European Organization for Research and Treatment of Cancer quality of life questionnaire. Higher scores represent better functioning. EORTC QLQ-BN20—The European Organization for Research and Treatment of Cancer quality of life questionnaire, brain tumor module. Higher scores represent a higher symptom burden. PHQ-9—patient health questionnaire-9. Higher scores indicate higher levels of depression. KPS—Karnowski performance scale. Higher scores represent better functioning. HVLT-R—Hopkins verbal learning test-revised. Higher scores represent better functioning. TMT-A—trail-making test, part A. Higher scores represent better functioning. TMT-B—trail-making test, part B. Higher scores represent better functioning. Verbal fluency test. Higher scores represent better functioning. Significant associations *p* < 0.05 indicated in bold numbers.

## Data Availability

The data is available from the corresponding author on reasonable request.
